# Interface engineering and defect passivation for enhanced hole extraction, ion migration, and optimal charge dynamics in both lead-based and lead-free perovskite solar cells

**DOI:** 10.1038/s41598-024-56246-4

**Published:** 2024-03-05

**Authors:** Muhammad Noman, Abdul Haseeb Hassan Khan, Shayan Tariq Jan

**Affiliations:** 1 U.S.-Pakistan Center for Advanced Studies in Energy, University of Engineering and Technology, Peshawar, Pakistan; 2 Department of Energy Engineering Technology, University of Technology, Nowshera, Pakistan

**Keywords:** Perovskite solar cell, BiI_3_, MAPbI_3_, MAGeI_3_, Passivation, Interface layer, Solar energy, Software

## Abstract

The study elucidates the potential benefits of incorporating a BiI_3_ interfacial layer into perovskite solar cells (PSCs). Using MAPbI_3_ and MAGeI_3_ as active layers, complemented by the robust TiO_2_ and Spiro-OMeTAD as the charge-transport-layers, we employed the SCAPS-1D simulation tool for our investigations. Remarkably, the introduction of the BiI_3_ layer at the perovskite-HTL interface significantly enhanced hole extraction and effectively passivated defects. This approach minimized charge recombination and ion migration towards opposite electrodes, thus elevating device performance relative to conventional configurations. The efficiency witnessed a rise from 19.28 to 20.30% for MAPbI_3_ and from 11.90 to 15.57% for MAGeI_3_. Additionally, MAGeI_3_ based PSCs saw an improved fill-factor from 50.36 to 62.85%, and a better J_sc_ from 13.22 to 14.2 mA/cm^2^, signifying reduced recombination and improved charge extraction. The FF for MAPbI_3_ based PSCs saw a minor decline, while the V_oc_ slightly ascended from 1.24 to 1.25 V and J_sc_ from 20.01 to 21.6 mA/cm^2^. A thorough evaluation of layer thickness, doping, and temperature further highlighted the critical role of the BiI_3_ layer for both perovskite variants. Our examination of bandgap alignments in devices with the BiI_3_ interfacial layer also offers valuable understanding into the mechanisms fueling the observed improvements.

## Introduction

In recent years, perovskite solar cells have made significant improvements in achieving power conversion efficiencies (PCE) to a peak of 25.7%^1^. The perovskite has emerged as a promising option in photovoltaic (PV) technologies owing to its exceptional light absorption characteristics^2–5^. The general formula for perovskite is ABX_3_, where “A” represents an organic/inorganic cation such as methylammonium (CH_3_NH_3_^+^, MA^+^)^6^ or formamidinium (NH = CHNH_3_^+^, FA^+^)^7^, the element “B” is characterized by the presence of metal cation such as lead (Pb^2+^), germanium (Ge^2+^) or tin (Sn^2+^) and “X” represents a halogen ion such as I^−^, Br^−^, or Cl^−^. The exceptional efficiency of solar cells utilizing three-dimensional ABX_3_ perovskite can be attributed to several key factors. These factors include their ability to absorb light across the visible to near-infrared spectrum, their minimal exciton binding energy (approximately 2 meV), direct band gap^8–11^, a large diffusion length, and high charge particle movement capability. These qualities make them highly favored as ideal photovoltaic materials^12^. Furthermore, reducing the defect density in perovskite films through various techniques has the potential to enhance photovoltaic performance, consequently increasing efficiency^13^. The unique characteristics of PSC position them as a compelling candidate for exploration within the domain of photovoltaic cells, among them the methyl ammonium lead tri-iodide (CH_3_NH_3_PbI_3_/MAPbI_3_) variant of PSC being particularly prevalent.

To successfully bring perovskite solar cells (PSCs) to the commercial market, it is essential to overcome significant challenges^14^. For instance, PSCs exhibit inadequate stability when exposed to temperature, humidity, and light. Moreover, the differential JV curves of PSCs are reliant on the scan directions, leading to the demonstration of the “hysteresis” phenomenon^15^. Efficient photo generation and enhancement of PCE are dependent on the optimization of hole and electron extracting from the absorber layer through appropriate selection of hole and electron transport layer. Recent research progress has significantly advanced the mitigation of hysteresis^16^ and the improvement of stability through the utilization of interfacial passivation techniques^17^.

Passivation involves applying a protective coating, typically a shielding material, through chemical interaction with the base substance, forming a micro-coating for protection^18^. The transition from an active to a passive state occurs through the formation of this passivation layer^19^. In the case of PSCs, passivation typically occurs in two forms: chemical passivation and physical passivation. Chemical passivation aims to minimize the presence of defect trap states, thus improving charge transfer across interfaces. Conversely, physical passivation involves the isolation of specific functional coatings from the surrounding environmental conditions to prevent cell degradation^20^. These passivation strategies are central to optimizing PSCs for enhanced PCE and overall performance.

Interface engineering between the perovskite and HTL is a well-established strategy for mitigating defects, effectively enhancing hole extraction by impeding secondary electrons^21^. The interface layer, situated between the absorber and the hole-transport layer, is an efficient technique to stop the prompt deterioration of perovskite layer caused by outside environment. Furthermore, it aids in mitigating recombination at interfaces, improving the cell’s efficiency in general as a result^22^. The interface engineering includes incorporation of supplementary interfacial layers (IL) between the active layer and HTL significantly enhances the efficiency of photovoltaic cells. In a recent study thiophene and pyridine compounds were introduced as the IL between the two layers, resulting in a noteworthy improvement in cell efficiency, increasing from 13 to 15.3%^23^. The implementation of passivation strategies has also led to a remarkable reduction in non-radiative recombination paths. In another study, employing the F4TCNQ as the IL achieved an even greater efficiency of 18%, compared to 15% without the IL. This enhancement was attributed to the increased electric field, which mitigated carrier losses occurring between the absorber layer’s surface and subsurface. The utilization of ILs creates an energy barrier that effectively hinders the recombination of photo-generated electrons in close proximity to the Perovskite/HTL interface^24^.

Studies have also explored the use of the halide perovskite layer as an IL. By adding an extra coating of MAPbI_3_ over the FAPbI_3_ absorber layer, they achieved an increase in efficiency of 16% from 14.5%. This increase was attributed to a significant boost in the conduction band minima at the interface, resulting in a higher V_oc_^25^. In another perovskite cell, the addition of an extra layer of FAPbBr_3−x_I_x_ over the (FAPbI_3_)_0.85_ (MAPbBr_3_)_0.15_ Perovskite absorber improved the PCE from 18.9 to 21.3% due to the additional coating effectively mitigated interfacial charge recombination, contributing to the enhanced performance^26^.

**Figure 1:**
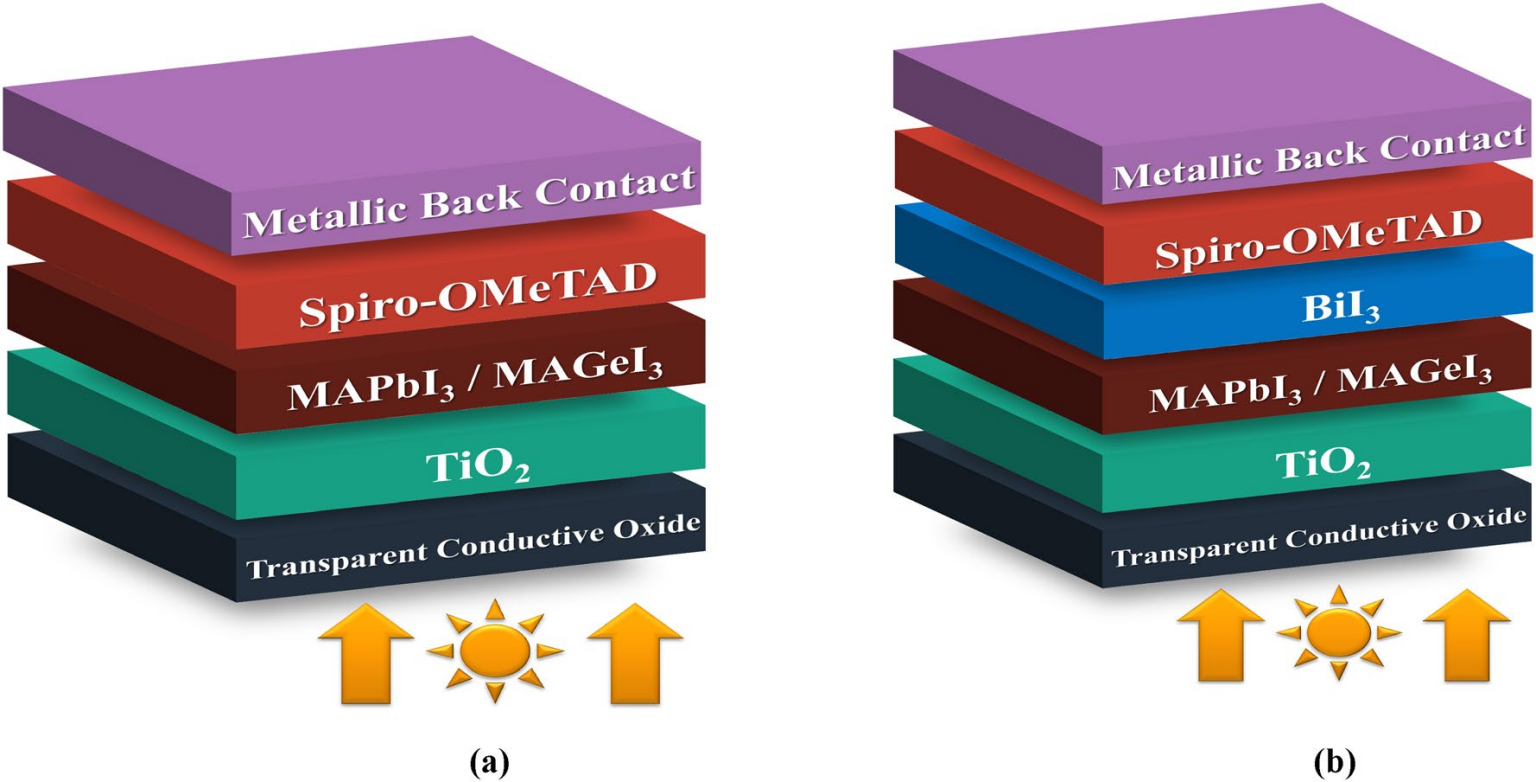
(**a**) Nip structure of PSC without IL, (**b**) PSC with interface layer.

Considering these findings, the utilization of bismuth (Bi) IL in perovskite devices holds promise for improving their performance. Bismuth iodide (BiI_3_) has gained attention due to its non-toxicity, low bandgap (1.72 eV), favorable absorption coefficients, and shorter carrier lifetimes, making it a suitable material for photovoltaic applications^27^. Bismuth used at the interface of HTL/Inorganic-Perovskite resulted in a notable enhancement in device efficiency in a study, increasing PCE from 7.4 to 11.9%. The Bi IL effectively prevents the movement of ions from the active layer, as demonstrated by these results^28^. According to the U.S. NMIC (National Mineral Information Centre) there exists a notable differences between the prices of Bismuth and Lead, with the Bismuth being comparatively lower^29^. In a study by Y.Hu pure BiI_3_ IL was deposited on the surface of the titanium oxide Electron Transport Layer, which led to an improvement in electron transport behavior, attributed to the neutralization of interface-associated trap states. Consequently, PCE increased significantly from 13.8% without the IL to 17.8% with the IL^30^. However, it’s worth noting that the amount of research conducted on the simulation and theoretical analysis of BiI_3_ ionic liquid-based systems at the interface between the absorber and Hole Transport Layer remains limited. In this investigation, an extra-thin BiI_3_ interface layer is introduced within the perovskite/HTL interaction as a passivation layer (Fig. 1b).

The primary function of the charge transport layers (CTL) is twofold: it collects electrons from the active layer and prevents their recombination with holes. TiO_2_ serves as the Electron Transport Layer (ETL) in our study due to its widespread use in PSC attributed to its favorable characteristics. Notably, TiO_2_ boasts excellent electron mobility, robust UV-light stability, and a high electron affinity, all of which facilitate efficient electron extraction and transport while minimizing recombination losses^31^. Beyond its exceptional performance, TiO_2_ is chemically stable, non-toxic, and abundantly available, making it an economical and eco-friendly choice for photovoltaic applications^32^. Similarly, spiro-OMeTAD serving as the HTL in this study, presents multiple advantages that make it highly preferred in both PSCs and solid-state dye-sensitized solar cells (ssDSCs). It exhibits high hole mobility, which is essential for efficient charge transport^33^, and its ionization potential aligns well with the perovskite absorber to facilitate effective charge extraction. Additionally, Spiro-OMeTAD can be synthesized and purified with high yield, making it cost-effective and environmentally stable compared to other hole transport materials (HTMs)^34^. Importantly, its chemical structure can be engineered to enhance thermal stability and extend operational lifespan^33^.

The PSC performance is influenced by several factors, including the thickness of the CTL as demonstrated by various experiments^35^. In this study, the optimized CTL thickness of 150 nm is adopted for both ETL and HTL. Previous experimental investigations have shown that an HTL thickness of 180 nm leads to superior photovoltaic (PV) efficiency compared to other tested thicknesses of 700, 500, 400, 450, 250, and 100 nm^36^. Beyond charge carrier transport and extraction, minimizing interface defects and ensuring the safety of the absorber layer are crucial for optimal device functionality. A thicker CTL can improve charge carrier extraction by more effectively reflecting them at the smooth interface of the HTL, which results in significant performance enhancements^37–39^.

In this study, we model two distinct PSC configurations: one without an IL while the other with an IL between the perovskite and HTL. A dual-focus approach is adopted for examining the performance enhancements in PSCs with two distinct absorber materials when integrated with a BiI_3_ layer. The first absorber is the well-established perovskite material of methylammonium lead iodide (MAPbI_3_) and the second is the newly emerging non-toxic perovskite of methylammonium germanium iodide (MAGeI_3_). A 40 nm thick BiI_3_ layer is utilized as the IL, which is sandwiched between the absorber and HTL. The selection of BiI_3_ is based on its remarkable ability to enhance hole extraction and ion migration, while also improving charge dynamics at the interface. This dual function is especially critical in both lead-based and lead-free PSCs. The solar cell simulator software SCAPS-1D was employed to analyze the beneficial effects of the BiI_3_ IL on device efficiency. Initial simulations used a standard n-i-p device without any IL to set a baseline. In the next step the BiI_3_ based IL was modelled into the cell structures. Results of the different PSCs were analyzed and compared in detail. The analysis performed in this study by using SCAPS-1D software provides a comprehensive understanding of how BiI_3_ IL influences key performance parameters by modifying absorber layer's doping concentration, operational temperature, and thickness. This study's innovative approach to investigating the band alignment, band offsets, charge transport, and recombination losses in the presence of a BiI_3_ IL adds valuable insight on PSC enhancement which are critical for advancing the field. Our findings suggest that the integration of a BiI_3_ IL is a promising strategy to elevate the performance of PSCs based on both MAPbI_3_ and MAGeI_3_. By presenting a focused and detailed examination of the BiI_3_ IL's impact, this work not only demonstrates its novelty but also establishes a benchmark for future research in enhancing the efficiency and stability of PSCs through sophisticated interface engineering techniques.

### Literature review

In the pursuit of advancing PSC technology, researchers have used various innovative interface engineering strategies to enhance both efficiency and stability. One significant breakthrough was achieved by Min et al., who developed PSCs with atomically coherent interlayers on SnO_2_ electrodes. This approach not only minimized interfacial defects but also optimized charge extraction and transport mechanisms. The result was a remarkable increase in PCE to 25.8%. By addressing the issue of interfacial defects, this study not only increased the PCE but also shed light on the critical role of electrode interlayer coherence in the operational stability and efficiency of PSCs^40^. In another study Zhang et al. introduced another layer of passivation by integrating bifunctional alkyl chain barriers at the crucial junction between perovskite and HTL. This effectively blocked electron recombination and protected the cells against moisture, leading to substantial increase in both efficiency and stability. The alkyl chain barriers represent a dual-function solution that not only enhances the electrical performance of PSCs but also addresses environmental durability, a key challenge for the commercial viability of perovskite-based photovoltaics^41^. Further exploring the potential of interface engineering, Dong et al. created interpenetrating interfaces between the perovskite layer and electron-transporting materials. This led to PSCs achieving efficiencies up to 22.2%, with significant improvements in operational stability and mechanical robustness. The addition of the interface layer highlights the importance of smooth connection between different layers in PSCs, ensuring efficient charge transport and reducing the risk of mechanical failure under operational stresses^42^. In another Chen et al. focused on the in situ formation of 2D perovskite layers at the interface of mixed perovskites and CuSCN. This method led to an increase in PCE from 13.72 to 16.75% while simultaneously improving moisture and photostability. The use of 2D perovskites as interface engineering layers highlights the versatile potential of these materials in enhancing both the efficiency and durability of PSCs, addressing two of the most critical challenges in the field^43^. Liu et al. demonstrated the significance of incorporating CsPbI_3_ quantum dots as an interface engineering layer, which enhanced the PCE from 15.17 to 18.56% and improved the stability of PSCs. This approach underscores the potential of quantum dot technologies in fine-tuning the optical and electrical properties of PSCs, offering a pathway to simultaneously achieve high efficiency and stability^44^. Kim et al. employed conformal quantum dot–SnO_2_ layers as electron transporters, achieving a PCE of 25.7%. This approach not only improved charge extraction but also underscored the potential of integrating quantum dots with traditional ETLs to push the limits of PSC efficiency^45^. Li et al. enhanced PSC performance through the modification of interfaces using a multifunctional fullerene derivative for TiO_2_ surface passivation. This method notably improved charge extraction, leading to a 20.7% improvement in PCE and highlighting the importance of surface passivation in achieving high-efficiency PSCs^46^. Salado et al. utilized thiazolium iodide for interface engineering, reducing thermal diffusion and significantly improving PCE. This strategy demonstrates the effectiveness of surface functionalization in enhancing both the efficiency and stability of PSCs, providing a promising route for future advancements^47^. Li et al. used interface ion exchange techniques to passivate surface defects, resulting in an extremely high open-circuit voltage of 1.19 V and an efficiency of 20.32%. This approach not only addresses surface defects but also opens new avenues for improving the photovoltaic performance of PSCs through ion exchange mechanisms^48^. Jiang et al. highlighted the excellence of SnO_2_ as an ETL, owing to its superior band alignment and high electron mobility. This technique is crucial for enhancing charge extraction, a key factor in the efficiency of PSCs, and points towards the potential of SnO in paving the way for the next generation of solar cells^49^.

Moving towards Bismuth materials, the study introduced Bismuth Telluride (Bi_2_Te_3_) nanoplates as an interlayer in all-inorganic PSCs, enhancing efficiency and stability. This interlayer, positioned between the CsPbBrI_2_ absorber layer and the Spiro-OMeTAD HTL, significantly reduced trap states and charge recombination. The optimized use of Bi2Te_3_ interlayer led to PCE increase from 7.46 to 11.96% and maintained over 70% of its initial PCE after 50 days without additional encapsulation, demonstrating an effective approach to improving PSC performance^41^. In another study, the incorporation of a BiI_3_ passivation layer between the compact TiO_2_ ETL and the perovskite absorber significantly enhances the efficiency and stability of planar perovskite solar cells. This interface engineering approach resulted in an increase in PCE from 13.85 to 16.15%, with a peak efficiency of 17.79%. The application of the BiI_3_ layer effectively facilitates electron extraction and minimizes hysteresis, marking a pivotal advancement in the performance optimization of perovskite solar cells^40^. Each of these studies collectively underscores the transformative impact of interface engineering on the development of PSCs and is summarized in Table 1. By optimizing the interfaces between various layers within PSCs, researchers have not only achieved significant efficiency and stability but also provided a roadmap for overcoming some of the most persistent challenges in the field of photovoltaic.

**Table 1:** Summary of discussed studies.

Absorber	Interface Layer	Interface Layer Position	CTL Layers	Efficiency Increased	References
FAPbI_3_	Cl-bonded SnO_2_ (coherent interlayers)	ETL/Perovskite	SnO_2_	From < 20 to 25.8%	^40^
MAPbI_3_	Bifunctional alkyl chain	HTL/Perovskite	Spiro-OMeTAD	Increased PCE more than 5%	^41^
Cs0.0_4_ (FA_0.84_MA_0.16_)_0.96_ Pb (I_0.84_Br_0.16_)_3_	FAI-incorporated SnO_2_ (FI–SnO_2_)	ETL/Perovskite	SnO_2_	Increased PCE to 22.2%	^42^
(FAPbI_3_)_0.88_(CsPbBr_3_)_0.12_	(5‐AVA)_2_ PbI_4_ (2D Perovskite Passivation layer)	HTL/Perovskite	CuSCN	From 13.72 to 16.75%	^43^
FAMAPbI_3_	CsPbX_3-_Quantum Dots	HTL/Perovskite	Spiro-OMeTAD	From 15.17 to 18.56%	^44^
FAPbI_3_	Polyacrylic acid-stabilized tin (IV) oxide	ETL/Perovskite	SnO_2_	Increased PCE to 25.7%	^45^
Perovskite	Fullerene Derivative (PCBB-2CN-2C8)	ETL/Perovskite	TiO_2_	Increased PCE to 20.7%	^46^
MAPbI_3_	Thiazolium Iodide	HTL/Perovskite	Various HTMs	Enhanced V_oc_ and fill factor	^47^
MAPbI_3_	Ion Exchange	ETL/Perovskite	SnO (ETL)	Increased PCE to 20.32%	^48^
MAPbI_3_	SnO_2_	ETL/Perovskite	SnO_2_	Increased PCE to 22%	^49^
CsPbBrI_2_	Bi_2_Te_3_	HTL/Perovskite	Spiro-OMeTAD	PCE of from 7.46 to 11.96%	^28^
MAPbI_3_	BiI_3_	ETL/Perovskite	TiO_2_	Increased PCE from 13.85 to 17.79%	^30^

Building on the foundation laid by different research in the field of PSC technology, the present study distinguishes itself through a focused investigation into the effects of a BiI_3_ interlayer on the performance of PSCs. Unlike other studies that have broadly explored interface engineering with various materials, this work focuses on the specific application of BiI_3_ ILs, providing a new insight in the optimization of PSC interfaces. This research differs from other work by applying a dual-focus approach of examining the performance enhancements in PSCs with two distinct absorber materials when integrated with a BiI_3_ IL. This study shifts away from the traditional focus on conventional materials such as SnO_2_, alkyl chain barriers, and quantum dots, which have been extensively explored for their roles in improving charge transport and addressing defects at interfaces. The targeted exploration of the BiI_3_ role as a passivation layer not only bridges a gap in the existing literature but also unveils a novel pathway for increasing the PSC efficiency through strategic interface modification.

## Device methodology

There are several numerical modeling software options accessible to facilitate the computational analysis of photovoltaic cell performance, including SETFOS, SCAPS, SILVACO, COMSOL, and ATLAS^50–53^. The choice of employing SCAPS-1D version 3.3.10 is driven by its beneficial attributes. These features include its open source nature, an intuitive interface that is easy to use and control, the capacity to simulate scenarios with or without light, and the capability to design a heterostructure-based system with up to seven layers^50,54,55^. The SCAPS-1D software is capable of assessing the effectiveness of photovoltaic through estimation of multiple parameters, such as PCE, FF, V_oc_, J_sc_, energy band, and IV Curve characteristics.

SCAPS-1D is based on solving the basic semiconductor equations that govern the operation of photovoltaic devices^56^. These equations include the Poisson’s Equation (Eq. 1), Continuity Equations (Eq. 2), Current Density Equations (Eq. 3) and Generation/Recombination (Eq. 4). The Poisson's Equation is a fundamental principle in electromagnetism and semiconductor physics, expressing the relationship between the electric potential in a region and the charge density within that region. It helps determine the electric field distribution across the semiconductor layers. The equation accounts for the static charge present and is crucial for understanding how electric fields form in response to charged defects, dopants, and the separation of electrons and holes within the device structure. While the Continuity Equations in semiconductor physics ensure the conservation of charge, describing how electron and hole densities change over time due to generation, recombination, and the flow of these carriers within the material. These equations are vital for predicting the dynamic behavior of charge carriers in response to external stimuli, such as light absorption in photovoltaic cells, and are essential for analyzing current flow and the effects of carrier recombination and generation on device performance. Similarly, the Current Density Equations describe how electrical current flows through a semiconductor material due to both the drift of charge carriers in an electric field and their diffusion from regions of high concentration to low concentration. These equations are key to modeling the transport of electrons and holes in photovoltaic devices, enabling the calculation of current–voltage characteristics under various conditions. They highlight the dual nature of charge transport, incorporating the effects of the material's electric field and the carriers’ thermal energy. Finally, the Generation/Recombination mechanisms show the processes by which charge carriers (electrons and holes) are created and finish within a semiconductor. Generation can occur through thermal energy or by absorbing photons, while recombination happens when electrons and holes combine, releasing energy. These mechanisms significantly impact the efficiency of photovoltaic devices, as they determine the net charge carrier density available for electrical current production. Understanding these mechanism is crucial for designing materials and device structures that minimize recombination losses and maximize generation for improved solar cell performance.

1 $$\nabla^{2} \phi = { } - \frac{\uprho }{\upvarepsilon }$$

2 $$\frac{dc}{{dt}} + \nabla \cdot J_{c} = G - R$$

3 $$J_{c} = qu_{c} c\nabla \phi \pm {\text{q}}D_{c} \nabla_{c}$$

4 $$R_{SRH} = \frac{{np - n_{i}^{2} }}{{{\uptau }_{n} \left( {n + n_{1} } \right) + {\uptau }_{p} (p + p_{1} }}$$

where ∇^2^ is the Laplacian operator, ϕ is the electric potential, ρ is the charge density, ε is the permittivity, c represent the electron (n) and hole densities (p), J_c_ are the current densities for electrons and holes, G is the rate of generation of carriers, R is the rate of recombination, q is the elementary charge, μ_c_ is the mobilities of electrons and holes, D_c_ is the diffusion coefficients for electrons and holes, ∇c is gradients of electron and hole densities, n_i_ is the intrinsic carrier density, τ_n_ and τ_p_ are the electron and hole lifetimes, and n_1_ and p_1_ are the electron and hole densities at thermal equilibrium, respectively.

**Figure 2:**
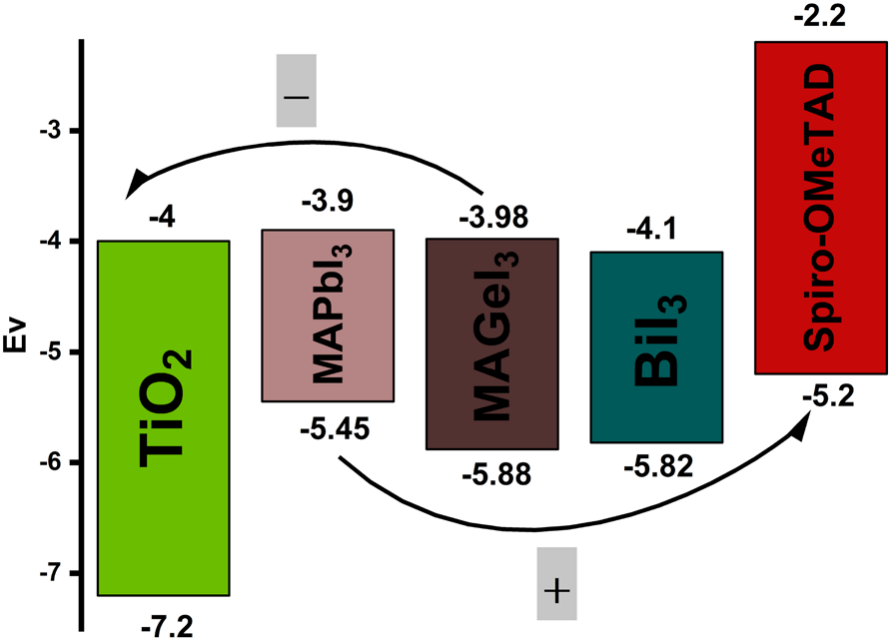
Energy level diagram.

Modeling the device in detail is a crucial step towards highlighting the impact of the IL on device functioning. It is intended to aid experimentalists in modifying their studies^57^. The simulations were conducted under standard testing conditions (STC) with a light intensity equivalent to AM 1.5 spectrums (1000 W/m^2^) and a temperature of 300 K. It is important to highlight that the simulations did not consider parasitic resistances. We have modeled four unique PSC structures, each utilizing ETL of TiO_2_ and HTL of Spiro-OMeTAD. Two of these structures employ MAPbI_3_ as the absorber layer. The remaining two structures utilize MAGeI_3_ as the absorber. One PSC from each absorber is modelled with the IL (Fig. 1b) while the other structure is without the IL (Fig. 1a). Figure 2 shows the energy level of the different materials used in this study. When exposed to light, photons impact the ETL and then disperse towards the HTL side. When photons are absorbed by the perovskite material, charge carriers are generated within the layer, which then migrate into the layers responsible for transporting electrons and holes. The optimized dimension of absorber layer, ETL, HTL, IL, along with various factor such as electron–hole mobility, effective density of states, doping densities, defect densities, and electron affinities have been collected from literature, which are comprehensively listed in Table 2^58–60^. This methodology allows us to systematically analyze the effects of incorporating an interface layer across different perovskite absorber materials.

**Table 2:** Modeling parameter of the layers used in PSC.

Parameters	TiO_2_	MAPbI_3_	MAGeI_3_	BiI_3_	Spiro-OMeTAD
Thickness (nm)	150	450	400	40	150
Bandgap (eV)	3.2	1.550	1.9	1.72	3.0
Electron affinity (eV)	4.00	3.9	3.980	4.1	2.2
Dielectric permittivity (relative)	10.00	6.5	10	5.780	3.0
CB effective density of states (cm^−3^)	2 × 10^18^	2.2 × 10^18^	1 × 10^16^	2.5 × 10^19^	2.2 × 10^18^
VB effective density of states (cm^−3^)	1.8 × 10^19^	1 × 10^18^	1 × 10^15^	2.5 × 10^19^	1.8 × 10^19^
Electron thermal velocity (cm/s)	1 × 10^7^	1 × 10^7^	1 × 10^7^	1 × 10^7^	1 × 10^7^
Hole thermal velocity (cm/s)	1 × 10^7^	1 × 10^7^	1 × 10^7^	1 × 10^7^	1 × 10^7^
Electron mobility (cm^2^/V.s)	2 × 10^1^	2	1.62 × 10^1^	6 × 10^2^	2 × 10^−4^
Hole mobility (cm^2^/V.s)	1 × 10^1^	2	1.010 × 10^1^	2 × 10^2^	2 × 10^−4^
Shallow uniform donor density N_D_ (cm^−3^)	1 × 10^17^	–	1 × 10^19^	1 × 10^16^	–
Shallow uniform acceptor density N_A_ (cm^−3^)	–	1 × 10^17^	1 × 10^19^	1 × 10^16^	1 × 10^17^
Defect type	Neutral	Neutral	Neutral	Neutral	Neutral
Capture Cross Section Electrons (cm^2^)	1 × 10^−15^	1.000 × 10^–15^	2 × 10^−14^	1 × 10^−15^	1 × 10^−15^
Capture Cross Section Holes (cm^2^)	1 × 10^−15^	1.000 × 10^–15^	2 × 10^−14^	1 × 10^−15^	1 × 10^−15^
Energetic Distribution	Single	Gaussian	Gaussian	Single	Single
Energy Level with Respect to Ev	0.6	0.60	0.65	0.6	0.6
Characteristic Energy (eV)	–	0.1 eV	0.1 eV	–	–
Total Defect Density Nt (cm^3^)	1 × 10^15^	1 × 10^14^	1 × 10^14^	1 × 10^15^	1 × 10^15^
Interface defects (cm^2^)	1 × 10^11^	–	–	1 × 10^11^	1 × 10^11^

## Results and discussion

### Effect of passivation layer on PSC energy band alignment

The performance of PSCs is significantly influenced by the energy band alignment between the PSC and the CTLs. For efficient electron extraction from the perovskite material, the conduction band (CB) of the ETL and the CB of the PSC must align with minimal offset. To block the holes their valence bands (VB) should show a considerable difference. If the VBs are too close, there is a risk that holes might migrate towards the ETL, leading to recombination. Proper alignment between the VB of the HTL and the perovskite material is crucial for facilitating hole separation. Similarly, a significant offset in their CBs is essential. If the CBs are aligned too closely, electrons may migrate towards the HTL, again leading to recombination.

The characterization of ideal band alignment in PSCs requires a minimal offset at the CB and a maximal offset at the VB between the perovskite and the ETL. The aim is to enable a smooth flow of electrons from the active layer to the ETL while simultaneously blocking hole transmission^61^. Likewise, the minimal valence band offset (VBO) and maximal conduction band offset (CBO) are crucial characteristics for both the HTL and perovskite material, facilitating seamless hole transmission from the absorber to the HTL while hindering electron mobility.

**Figure 3:**
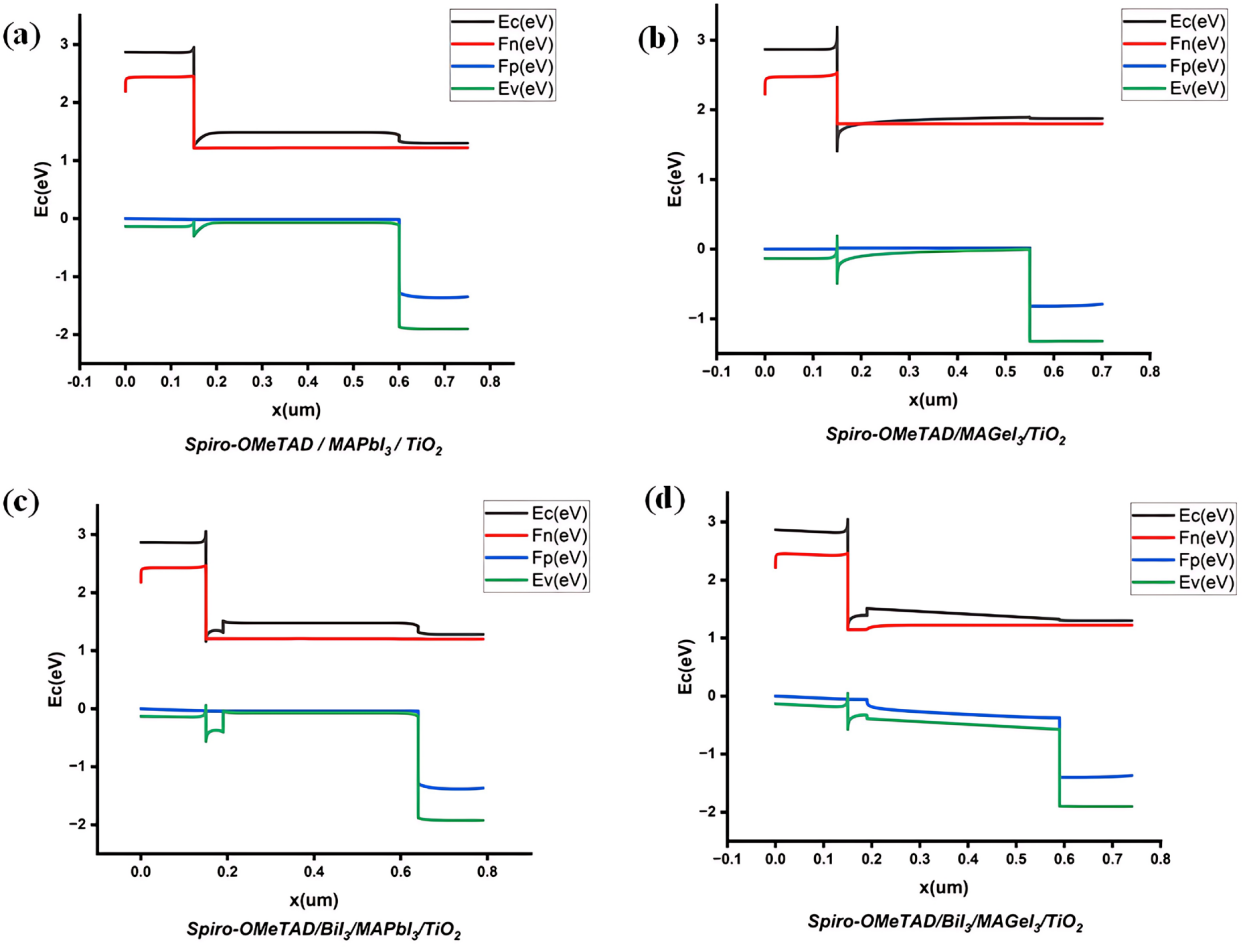
MAPbI_3_ and MAGe_3_ band alignment with and without a BiI_3_ layer.

The engineering of an IL between the absorber and the HTL is a widely recognized approach for effectively mitigating defects, typically enhancing hole extraction by impeding the movement of secondary electrons^21^. Figure 3 shows the energy band alignment of the PSCs while Table 3 shows the VBO and CBO formed by the layers. The VBO and CBO have been calculated from the electron affinity (χ) and band gap (Eg) of the material using the formula:

5 $${\text{CBO}} = \left( {\chi_{{{\text{Per}}}} {-}\chi_{{{\text{CTL}}}} } \right)$$

6 $${\text{VBO}} = \left( {\chi_{{{\text{CTL}}}} {-}\chi_{{{\text{Per}}}} + {\text{Eg}}_{{{\text{CTL}}}} {-}{\text{Eg}}_{{{\text{Per}}}} } \right)$$

**Table 3:** VBO and CBO on interfaces.

Interface	CBO (eV)	VBO (eV)
Perovskite/ETL		
MAPbI_3_/TiO_2_	− 0.1	1.75
MAGeI_3_/TiO_2_	− 0.02	1.32
Perovskite/HTL		
MAPbI_3_/spiro-OMeTAD	1.7	− 0.25
MAGeI_3_/spiro-OMeTAD	1.78	− 0.68
Perovskite/passivation		
MAPbI_3_/BiI_3_	− 0.2	0.37
MAGeI_3_/BiI_3_	− 0.12	− 0.06
Passivation/HTL		
MAPbI_3_/BiI_3_/spiro-OMeTAD	1.9	0.62
MAGeI_3_/BiI_3_/spiro-OMeTAD	1.9	0.62

The introduction of an IL has been observed to adjust the alignment of energy levels among the films and prevent ion migration. The rate of hole injection, especially between the active layer and the HTL, is influenced by the alignment of the interface energy levels. The presence of an energy barrier at the interfaces leads to charge carrier recombination and thus limits the efficiency of charge transfer. Conversely, the absence of an EB across the interface facilitates efficient charge transfer and injection, reducing recombination rates. The incorporation of a BiI_3_ IL at the junction between the absorber and the HTL enhances hole transport across the interface and may help mitigate interface charge recombination.

In the context of the MAPbI_3_/TiO_2_ interface, as delineated in Table 2, a CBO of − 0.1 eV and a VBO of 1.75 eV facilitate efficient the transport of charge from MAPbI_3_ to the TiO_2_. The small CBO facilitates electron flow, while the substantial positive VBO impedes hole migration into the TiO_2_ layer, thereby minimizing electron–hole recombination at this juncture.

At the absorber/HTL interface, with a CBO of 1.7 eV and a VBO of − 0.25 eV, there is a promotion of efficient hole transport from MAPbI_3_ to Spiro-OMeTAD due to the small VBO and large CBO. The large CBO serves as an electron-blocking layer, preventing electrons from reaching the Spiro-OMeTAD layer thereby lowering the likelihood of recombination.

Introduction of the BiI_3_ interfacial layer between the active layer and HTL provides a climbing ladder for the holes to the HTL. The IL forms a CBO of − 0.2 eV and a VBO of 0.37 eV at the MAPbI_3_/BiI_3_ interface. The VBO of 0.37 forms a spike which increases the electric potential at the heterojunction than the MAPbI_3_/HTL hetero junction which forms a cliff. The higher electric potential efficiently transfers holes from MAPbI_3_ to BiI_3_, reducing the likelihood of hole recombination. At the Spiro-OMeTAD/BiI_3_ interface, the CBO of 1.9 eV produces a larger barrier for the electrons.

Similarly, at the MAGeI_3_/TiO_2_ interface, a CBO of − 0.02 eV and a VBO of 1.32 eV support the efficient electron transport from MAGeI_3_ to TiO_2_. The small CBO of MAGeI_3_ with TiO_2_, along with a large positive VBO, promotes the flow of electrons while restricting hole migration to TiO_2_, thus reducing recombination at this interface.

At the MAGeI_3_/HTL interface, a CBO of 1.78 eV and a VBO of − 0.68 eV is formed. The large CBO blocks electrons to the HTL. However, the large negative VBO forms a cliff which not only blocks some holes but also reduces the built-in potential. Upon introducing a BiI_3_ interface layer between MAGeI_3_ and Spiro-OMeTAD, the energy level alignment features a CBO of − 0.12 eV and a VBO of − 0.06 eV. The small VBO forms a ladder for the holes to climb to reach the HTL. Furthermore, the significantly small cliff causes a higher electric potential at the hetero junction which improves hole transportation.

In conclusion, the strategic introduction of a BiI_3_ IL has demonstrated a tangible impact on the charge transport dynamics within perovskite solar cells. By fine-tuning the energy level alignment across the interfaces, the BiI_3_ IL optimizes the transport of charge carriers for both perovskites. The IL not only facilitates as a ladder for holes towards the HTL but also increases the electric potential at the interface.

### IV characteristics

**Figure 4:**
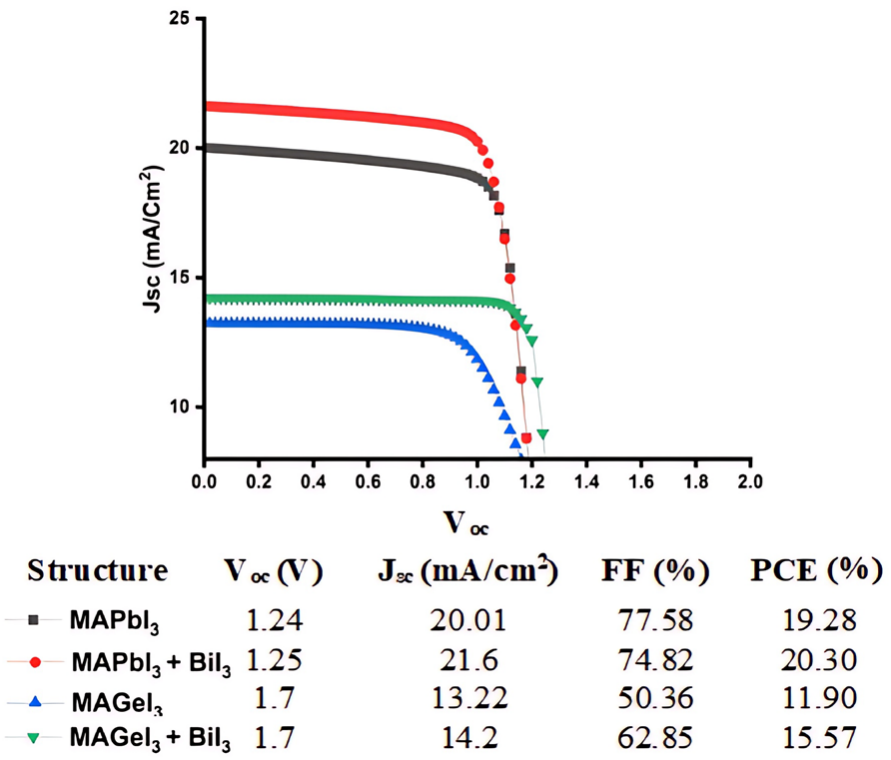
The current–voltage (I–V) characteristic curves of photovoltaic devices both with and without a BiI_3_ interface layer.

Comprehensive IV characteristics derived from the four structuresare are shown in Fig. 4. The TiO_2_/MAPbI_3_/Spiro-OMeTAD exhibited a PCE of 19.28%, J_sc_ of 20.01 mA/cm^2^, FF of 77.58%, and a V_oc_ of 1.24 V. Similarly, in the case of TiO_2_/MAGeI_3_/Spiro-OMeTAD configuration, the simulated device demonstrated a V_oc_ of 1.7 V, a J_sc_ of 13.22 mA/cm^2^, a FF of 50.36%, and a PCE of 11.90%. The structures were then analyzed with the passivation layer. Remarkably, the introduction of this BiI_3_ interface led to a notable enhancement in key photovoltaic parameters. In MAPbI_3_, the V_oc_ observed a shift from 1.24 to 1.25 V. Concurrently, the J_sc_ displayed an improvement, transitioning from 20.01 to 21.6 mA/cm^2^. These increments culminated in the rise of the overall PCE from 19.28 to 20.30%. These enhancements contributed to an overall PCE increase from 19.28 to 20.30%. This improved performance is because of the favorable band alignment facilitated by the BiI_3_ interlayer, which optimizes charge carrier separation and extraction, as depicted in Fig. 3. The optimized band alignment reduces charge carrier recombination at the interface and promotes more efficient charge transport and extraction, crucial for achieving higher PCE.

Similar to this, by the introduction of BiI_3_ interface between the MAGeI_3_ absorber and Spiro-OMeTAD HTL the V_oc_ remained invariant at 1.7 V. Yet, J_sc_ witnessed an increased from 13.22 to 14.2 mA/cm^2^. The FF increased significantly from 50.36 to 62.85%. Consequently, the PCE jumped from 11.90 to 15.57% with BiI_3_ integration. The enhancement in J_sc_ and FF, and thereby the PCE, is directly linked to the advantageous band alignment introduced by the BiI_3_ layer. This alignment reduces charge carrier recombination and improves interfacial impedance, facilitating more efficient charge transport across the interfaces. The presence of the BiI_3_ layer acts as a bridge, enhancing charge flow between the absorber and HTL, which is critical for the efficient extraction of photo-generated carriers. These results show the important role of interface engineering, particularly through the integration of passivation layers like BiI_3_, in enhancing the photovoltaic performance of PSCs. By optimizing the interfacial properties and band alignment, significant improvements in key performance parameters such as J_sc_, FF, and PCE can be achieved, paving the way for the development of more efficient and stable PSCs.

Table 4 compares the results of this study with fabricated experimental data of MAPbI_3_ PSC using interface layers. When Thiophene and Pyridine are used as the passivation layer in the PSC the PCE of the cells increase from 13 to 15.3% and 16.5%, respectively. This improvement is attributed to the passivation of under-coordinated Pb ions within the perovskite crystal. Similarly, when Tetrafluoro-tetracyanoquinodimethane (F4TCNQ) is used as the interface layer the PCE increases from 14.3 to 16.4% and improved long-term stability in ambient air. When the Bismuth based layer of Bi_2_Te_3_ is used as the passivation layer, the PCE increases from 7.46 to 11.96% and maintained over 70% of its initial PCE after 50 days without additional encapsulation. Lastly, the integration of a BiI_3_ passivation layer, the PCE increases from 13.85 to 16.15%, highlighting the layer's effectiveness in facilitating electron extraction and minimizing hysteresis. Similarly in our work when the BiI_3_ is used as the passivation layer, the PCE of MAPbI_3_ increases by 1.02% while for MAGeI_3_ it increases by 4.63%. The simulation models produce results that align closely with the experimentally fabricated data, underscoring the predictive accuracy and relevance of our computational approach in mirroring real-world PSC performance enhancements.

**Table 4:** Comparative impact of passivation layers on the PCE of perovskite solar cells.

Perovskite	Interface layer	PCE increase	References
MAPbI_3_	Thiophene	From 13 to 15.3%	^23^
MAPbI_3_	Pyridine	From 13 to 16.5%	^23^
MAPbI_3_	F4TCNQ	From 14.3 to 16.4%	^24^
MAPbI_3_	Bi_2_Te_3_	From 7.46 to 11.96%	^28^
MAPbI_3_	BiI_3_	From 13.85 to 16.15%	^30^
MAPbI_3_	BiI_3_	From 19.28 to 20.3%	This work
MAGeI_3_	BiI_3_	From 11.9 to 15.57%	This work

### Impact of absorber layer thickness on photovoltaic parameters

**Figure 5:**
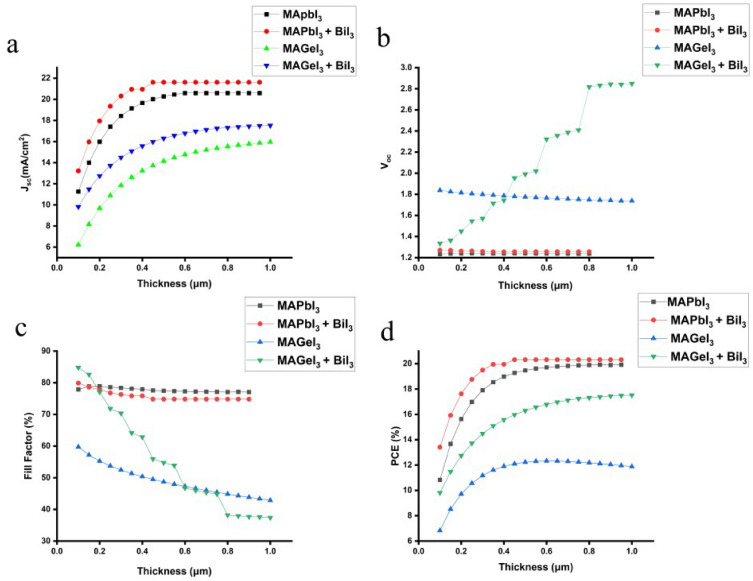
Impact on J_sc_, V_oc_, FF and PCE while changing absorber thickness in presence and absence of BiI_3_ IL.

The thickness of the active layer plays a critical role in affecting the optical properties, morphology, and overall performance of PSC^62,63^. Figure 5 shows the effect of absorber thickness on the PSCs. Increasing the thickness of both MAPbI_3_ and MAGeI_3_ layers influences the device performance, particularly when a BiI_3_ interfacial layer is introduced. It's observed that for both types of structures, the J_sc_ rises as the layer thickness increases, due to enhanced light absorption capabilities^64,65^. Larger thickness leads to absorption of more photons, especially those of higher wavelength. These photons contribute to more photogeneration of charge carriers which leads to higher J_sc_. Specifically, for the MAPbI_3_ and MAGeI_3_ with the BiI_3_ IL consistently outperform their counterparts in terms of J_sc_ . The improvement is attributed to the BiI_3_ layer acting as a passivation interface, which facilitates efficient charge extraction and reduces non-radiative recombination losses^66^.

For MAPbI_3_ devices equipped with a BiI_3_ interlayer, an initial decline in the V_oc_ is observed as the layer thickness increased, which then stabilized. This behavior is attributed to the initial reduction in charge carrier recombination rates facilitated by the BiI_3_ layer. The BiI_3_ layer acts as a barrier, impeding non-radiative recombination pathways at the interface, which initially lowers V_oc_ due to the adjustment phase of charge carriers to the new interface dynamics. As the thickness increases, the effect of the BiI_3_ layer in reducing recombination becomes more prominent, leading to stabilization of V_oc_. The reduction in charge recombination is a critical factor in stabilizing V_oc_, as it allows for more efficient charge separation and extraction, ultimately enhancing device performance. Conversely, in the absence of a BiI_3_ interlayer, MAPbI_3_ devices exhibited fluctuating V_oc_ values with a general decline at greater thicknesses. This decline is directly linked to increased trap-assisted recombination. Without the passivating effect of the BiI_3_ layer, charge carriers are more susceptible to recombination through defect states within the perovskite layer, exacerbated as the layer thickness increases, leading to a decrease in V_oc_. For devices based on MAGeI_3_ with a BiI_3_ interlayer, a significant improvement in V_oc_ was observed with increasing thickness. This improvement is due to the enhanced energy band alignment between the HTL, the BiI_3_ interface, and the absorber layers. The optimized band alignment facilitates more effective charge separation and minimizes recombination losses, directly contributing to the observed increase in V_oc_. In contrast, without the BiI_3_ interlayer, the V_oc_ for MAGeI_3_ devices decreased with increased thickness, which is attributed to enhanced bulk recombination, diminishing the quasi-Fermi level separation and thereby reducing the device's overall efficiency.

The FF trends for both MAPbI_3_ and MAGeI_3_ solar cells, irrespective of the presence of a BiI_3_ interlayer, showed a decreasing pattern with increasing layer thickness. This phenomenon is attributed to increased bulk recombination and the accompanying resistive challenges that rise in series resistance with thicker layers. In the specific case of MAPbI_3_, the initial presence of a higher FF upon the introduction of a BiI_3_ interlayer is linked to a higher VBO, which stabilizes at higher thickness, showcasing the BiI_3_ layer's role in mitigating recombination and stabilizing device performance^67^.

The PCE trends observed for both MAPbI_3_ and MAGeI_3_ solar cells further underscore the pivotal role of the BiI_3_ interlayer. The enhanced PCE in MAPbI_3_ devices with BiI_3_ is a direct result of improved photovoltaic parameters, such as J_sc_ and V_oc_, which are in line with the previously discussed V_oc_ and FF observations^63^. The increase in PCE with layer thickness in both cell types is attributed to improved light absorption and charge generation within the thicker perovskite layers. However, the MAGeI_3_ devices with BiI_3_ consistently exhibit superior performance, benefiting from the synergistic improvements in FF, J_sc_, and V_oc_. These improvements are indicative of the BiI_3_ interlayer's efficacy in optimizing charge transfer dynamics and enhancing overall PV cell performance, thereby offering a comprehensive understanding of the physical mechanisms at play^66^.

### Impact of absorber layer doping on photovoltaic parameter

**Figure 6:**
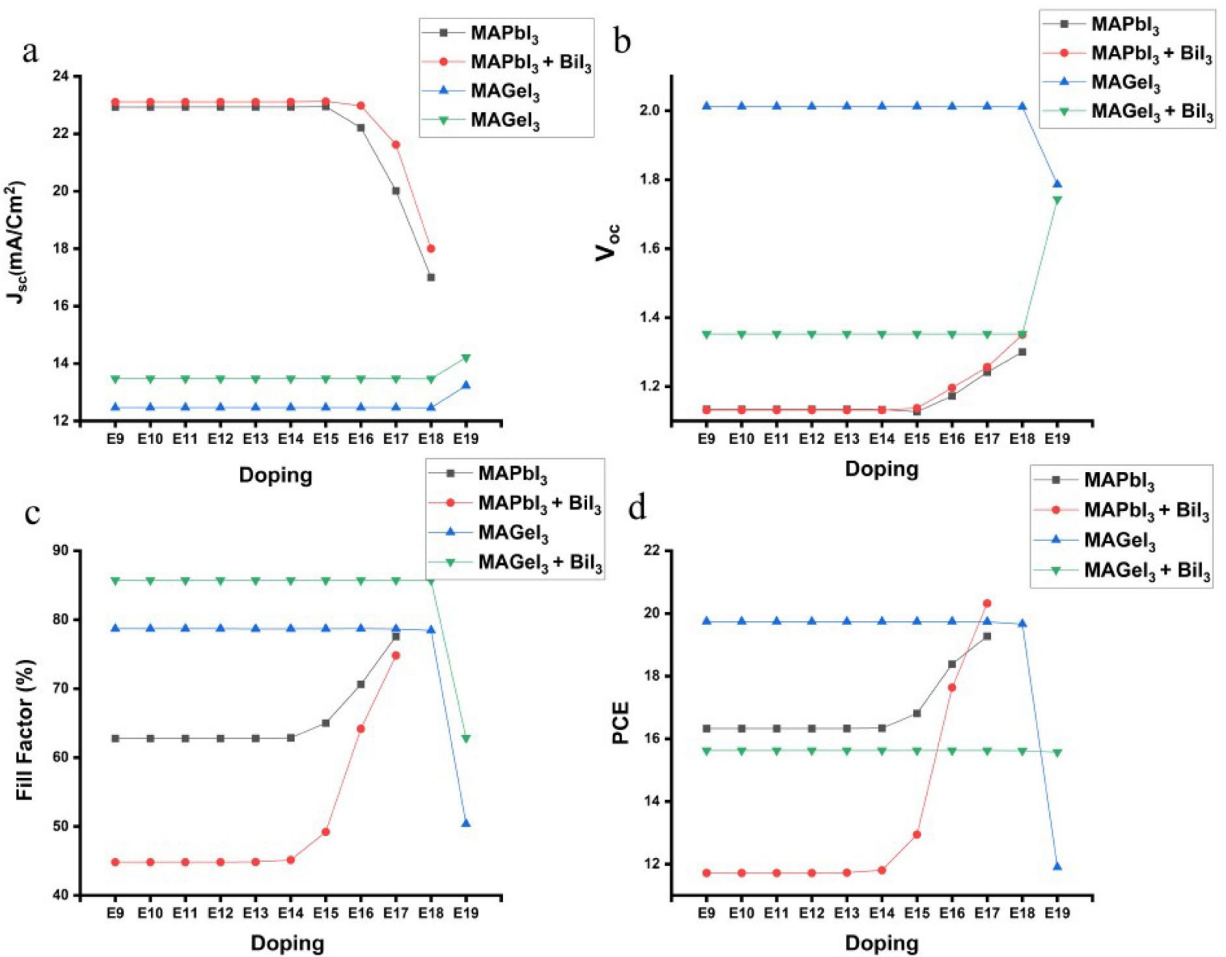
Impact on J_sc_, V_oc_, FF and PCE while changing absorber doping in presence and absence of BiI_3_ IL.

In perovskite solar cells, the absorber layer plays a critical role in light absorption and charge generation^68,69^. The strategic introduction of n-type or p-type dopants, a process known as doping, serves to enhance the cells' photovoltaic efficiency by fine-tuning charge transport and carrier density, thereby optimizing device performance^70^. Figure 6 shows the impact of doping concentrations within the absorber layer on PSC performance, showcasing that both MAPbI_3_ and MAGeI_3_ cells exhibit variable J_sc_ levels in response to altered doping levels^71^. Notably, MAPbI_3_ cells equipped with a BiI_3_ interface layer consistently surpass their counterparts, particularly when doping concentrations are optimized, a trend also observable in MAGeI_3_ cells. Excessive doping, however, can induce increased recombination rates and diminish J_sc_, though the presence of BiI_3_ effectively mitigates these negative effects by preserving elevated J_sc_ values.

Further analysis reveals the effects of doping variations on the V_oc_ for both MAPbI_3_ and MAGeI_3_ configurations, with and without a BiI_3_ interface layer. Initially, MAPbI_3_ cells lacking a BiI_3_ layer exhibited slightly enhanced V_oc_ within a lower doping range (E9–E14 cm^−3^) due to reduced parasitic resistance. Beyond this range, V_oc_ values for MAPbI_3_ cells with a BiI_3_ layer began to exceed those without, attributed to the BiI_3_ layer's passivation effects, which curtail recombination losses at higher doping levels (E15–E17 cm^−3^). This upward trend in V_oc_, facilitated by the BiI_3_ interface, persisted across further doping levels, underscoring the layer's critical role in harmonizing charge extraction and recombination loss mitigation. In contrast, MAGeI_3_ cells initially favored configurations without BiI_3_, yet V_oc_ remained relatively constant across a broad doping spectrum (E9–E18 cm^−3^) for both setups, suggesting suboptimal doping levels. A significant shift was observed at higher doping levels, where MAGeI_3_ cells without BiI_3_ experienced a notable drop in V_oc_, whereas those with BiI_3_ saw a substantial increase, highlighting the interface layer's effectiveness in reducing recombination losses and enhancing charge extraction.

The FF dependence on doping variation was also scrutinized for both MAPbI_3_ and MAGeI_3_ cells, revealing that the absence of BiI_3_ initially resulted in a higher FF for MAPbI_3_ cells, a phenomenon linked to increased series resistance with the BiI_3_ layer. As doping increased, FF remained relatively stable for both cell types until the optimal doping level of E14–E15 cm^−3^ was reached, beyond which the FF of MAPbI_3_ cells without BiI_3_ continued to rise, albeit more modestly compared to those with the BiI_3_ layer, which benefited from diminished recombination losses at elevated doping levels. MAGeI_3_ cells exhibited stable FF up to E18 cm^−3^, after which those without BiI_3_ suffered a significant FF decrease, in contrast to the less severe reduction observed in cells with BiI_3_, emphasizing the layer's role in improving charge transport and minimizing series resistance at optimized doping levels.

The PCE trends also reflect the relation between doping levels and the presence of a BiI_3_ interface layer. Initially, MAPbI_3_ cells without BiI_3_ showcased higher PCE values due to reduced parasitic resistance. However, a dramatic shift was noted at higher doping levels (E14 cm^−3^ and E17 cm^−3^), where the PCE of MAPbI_3_ cells with BiI_3_ surged, benefiting from synergistic improvements in V_oc_, FF, and reduced recombination losses, thereby outperforming those without the interface layer. Similarly, MAGeI_3_ cells initially exhibited higher PCE without BiI_3_, but this advantage dwindled at elevated doping levels, where the presence of BiI_3_ either stabilized or slightly increased PCE, attributed to enhanced charge extraction and minimized recombination losses. These observations highlight the critical importance of doping optimization and the integration of a passivation interface layer for advancing the efficiency and stability of PSCs, especially at higher doping concentrations.

### Effect of temperature variation on PSC

**Figure 7:**
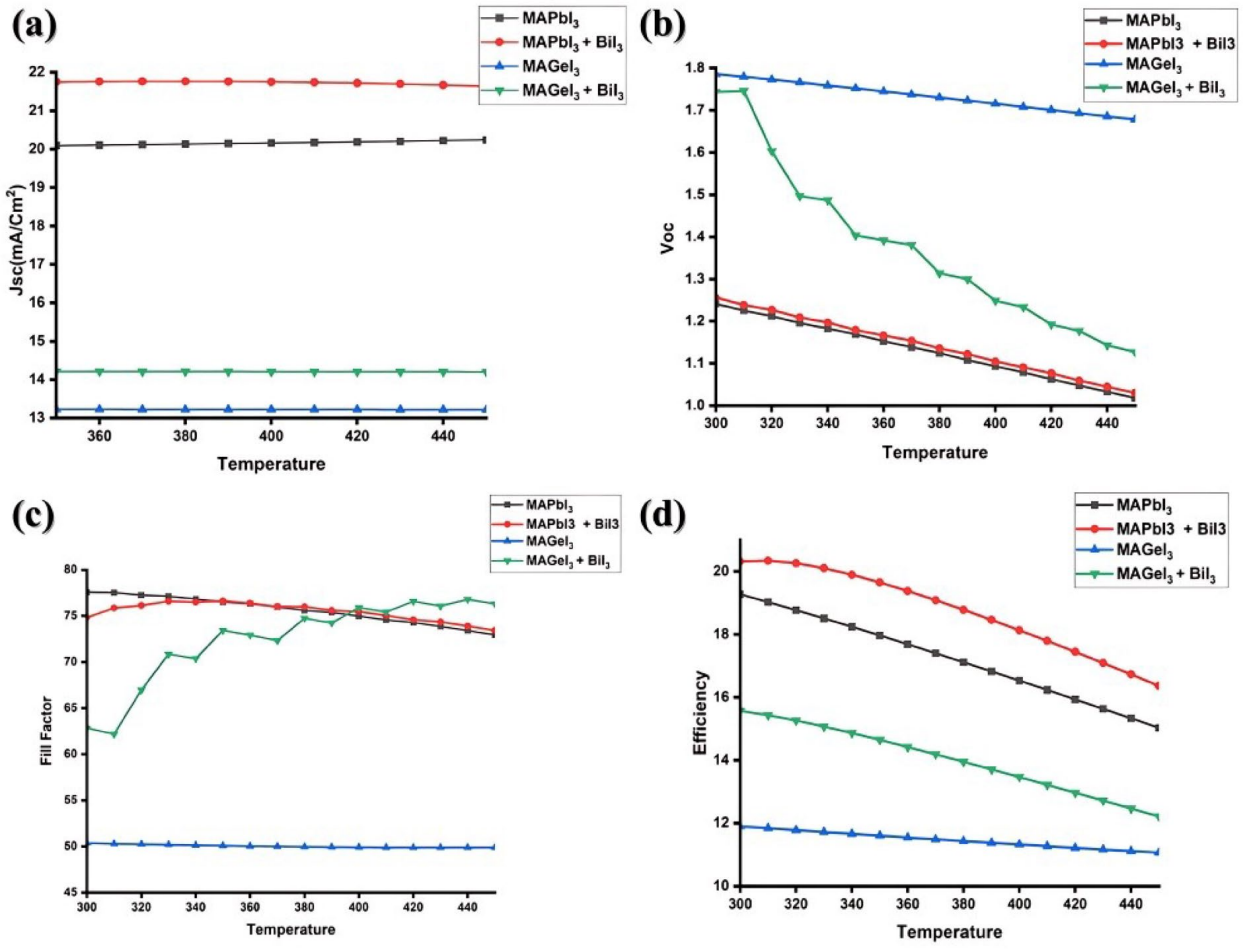
Effect of temperature variation on MAPbI_3_ and MAGeI_3_ on with and without BiI_3_ interface layer.

In the quest for enhanced efficiency and thermal stability in perovskite solar cells, this study delves into simulations comparing the performance of these cells with and without interface layers, elucidating the implications of temperature on each parameter. Temperature has a notable influence on how well solar cells function^11,72^. Most PV cells achieve their highest efficiency when operating at around room temperature, which is about 300 K. To study how temperature affects the performance of PSC, the temperature varied across the range of 300–450 K for all the structures and the results are presented in Fig. 7. The efficiency of PV cells declines with an increase in temperature. Crystalline silicon cells, a temperature coefficient ranging from − 0.3 to − 0.5%/°C is usual^73^. This means that for each degree Celsius rise in temperature, the efficiency of the solar cell decreases by that percentage. The fundamental physics underlying the temperature effect on PSCs begins with the thermal dependency of the semiconductor bandgap. As temperature increases, the bandgap of the semiconductor material narrows due to the increased vibrational energy of the lattice^74^. This reduction in bandgap energy directly leads to a decrease in V_oc_, as the potential difference that can be generated by the solar cell is diminished. The narrowing bandgap reduces the energy barrier for charge carrier recombination, thereby increasing non-radiative recombination rates and further decreasing V_oc_. Furthermore, the increase in temperature also leads to an increase in the intrinsic carrier concentration, which further reduces the open-circuit voltage. The Perovskite solar cells, which have gained attention due to their impressive lab-scale efficiencies, demonstrate a complex relationship with temperature, often degrading faster at elevated temperatures^75^. Some perovskite structures undergo phase transitions when subjected to elevated temperatures, such as the shift from tetragonal to cubic phases in organic–inorganic lead halide perovskites^76^. This can lead to a change in their optical and electronic properties^77^. For instance, phase changes can affect the material's absorbance and its electronic band structure, potentially degrading the cell's efficiency and thermal stability. One of the key concerns with perovskite materials is their thermal stability. Prolonged exposure to high temperatures can lead to degradation of the material, significantly reducing its efficiency and lifespan^78^. Temperature variation significantly affects the performance of perovskite-based solar cells. An increase in temperature can cause a decline in open-circuit voltage and fill factor, leading to a reduction in overall efficiency^79^. Increased temperature also impacts both the bandgap energy and the material's conductivity within the cell, leading to a decline in its overall performance^80^.

Analyzing V_oc_ variations in Fig. 7, MAPbI_3_ systems displayed a consistent decline with increasing temperature, both in the presence and absence of the BiI_3_ interface layer. The reduction in V_oc_ can be scientifically rationalized by the increased non-radiative recombination rates at higher temperatures. The energy difference between the Fermi levels of the electron and HTL might reduce, leading to a decrease in V_oc_. For MAGeI_3_ systems without the interface, a similar decline in V_oc_ was noted with rising temperature, potentially for the same reason. However, when paired with the BiI_3_ interface layer, MAGeI_3_ devices exhibited a more pronounced V_oc_ drop.

In the context of J_sc_, it was observed that as temperature escalated, MAPbI_3_ devices, regardless of the presence of the BiI_3_ interface layer, showed negligible variations. This can be attributed to the fact that J_sc_ is predominantly dependent on the number of photo-generated carriers, and in the MAPbI_3_ system, temperature elevation might not substantially affect this number or the carrier collection efficiency. Similarly, in MAGeI_3_-based systems, both with and without the interface layer, J_sc_ remained relatively unaltered with temperature changes. This indicates that the intrinsic properties and electronic pathways of MAGEI_3_ might be relatively resistant to temperature perturbations.

Considering the fill factor, temperature rise caused a decline in FF for MAPbI_3_ devices without the interface. In contrast, devices with the BiI_3_ interface layer displayed a marginal increase in FF at higher temperatures. The rise can be explained by the possibility that the BiI_3_ layer optimizes charge transport or minimizes series resistance under such conditions. On the other hand, MAGEI_3_-based devices exhibited stable FF values across temperature variations. However, incorporating the BiI_3_ interface layer resulted in an enhanced FF as temperature surged. This increase could stem from the synergistic interplay between MAGEI_3_ and BiI_3_, potentially enhancing charge extraction or reducing recombination at the interface.

Lastly, efficiency assessments of both MAPbI_3_ and MAGEI_3_ systems, regardless of the BiI_3_ interface's presence, unveiled a decline with rising temperatures. This is a holistic outcome of the combined impact on J_sc_, V_oc_, and FF, and resonates with the commonly understood behavior that elevated temperatures often deteriorate the performance metrics of many photovoltaic materials.

## Conclusion

In conclusion, our detailed study highlights the benefits of introducing a Bismuth Iodide (BiI_3_) interlayer (IL) at the interface between the absorber and the HTL. Two different perovskites of MAPbI_3_ and MAGeI_3_ are used alongside Titanium Dioxide (TiO_2_) as the ETL and Spiro-OMeTAD as HTL. Utilizing the SCAPS-1D simulation tool, we were able to clarify the mechanisms that contribute to the improved efficiency resulting from the BiI_3_ integration. Notably, simply adding a BiI_3_ layer at the perovskite-HTL interface significantly improves hole extraction by effectively reducing defect states, which in turn lowers charge recombination and ion migration. This strategic addition results in better device performance compared to traditional setups. The PCE of both MAPbI_3_ and MAGeI_3_ PSCs saw a considerable increase, showcasing the potential of BiI_3_ IL for practical PSC applications. The efficiency witnessed a rise from 19.28 to 20.30% for MAPbI_3_ and from 11.90 to 15.57% for MAGeI_3_. Additionally, MAGeI_3_ based PSCs saw an improved fill-factor from 50.36 to 62.85%, and a better J_sc_ from 13.22 to 14.2 mA/cm^2^, signifying reduced recombination and improved charge extraction. The FF for MAPbI_3_ based PSCs saw a minor decline, while the V_oc_ slightly ascended from 1.24 to 1.25 V and J_sc_ from 20.01 to 21.6 mA/cm^2^. Additionally, a thorough analysis of temperature variations revealed interesting findings. It was observed that while the performance of MAPbI_3_-based devices remained relatively stable with temperature changes, regardless of the BiI_3_ interface layer, the efficiency of both MAPbI_3_ and MAGeI_3_ compositions decreased with rising temperatures. These temperature dependencies highlight the crucial role of the BiI_3_ interface layer in not only adjusting charge dynamics but also in reducing the negative impacts of thermal stress on overall device performance. Detailed evaluations of layer thickness and doping gradients using SCAPS-1D reinforce the idea that the presence of BiI_3_ IL is crucial for both performance and durability across perovskite structures. Although the inclusion of BiI_3_ slightly increased the optimized thickness of MAPbI_3_, it was much more significant for MAGeI_3_. The optimized thickness increased from 0.4 to between 0.8 and 1 μm. While for doping, the V_oc_ sees significant increase in structures having the BiI_3_ as IL, especially from E15 to E17 cm^−3^, emphasizing the interface's role in balancing charge extraction and recombination losses. By reducing defect states and limiting recombination pathways, and providing resistance against temperature variations, the BiI_3_ layer could play a significant role in addressing stability issues while also enhancing PSC performance metrics. Overall, our results suggest that the strategic addition of a BiI_3_ interfacial layer within MAGeI_3_ and MAPbI_3_-focused PSCs marks a significant advancement in sustainable energy conversion technologies, especially in settings with notable temperature variations.

## Data Availability

Data available upon reasonable request from the corresponding author Dr. Muhammad Noman (muhammad.noman@uetpeshawar.edu.pk).

## References

[1] Prathapani S, Bhargava P, Mallick S (2018). Electronic band structure and carrier concentration of formamidinium–cesium mixed cation lead mixed halide hybrid perovskites. Appl. Phys. Lett..

[2] Qiu J (2019). Toward a new energy era: Self-driven integrated systems based on perovskite solar cells. Solar RRL.

[3] Jan ST, Noman M (2024). Exploring the potential of MAGeI3 perovskite cells with novel charge transport material optimization. Optik.

[4] Jan ST, Noman M (2023). Comprehensive analysis of heterojunction compatibility of various perovskite solar cells with promising charge transport materials. Sci. Rep..

[5] Khan, A. H. H. *et al.*, Exploring the efficiency and transparency in toxic and non-toxic perovskite solar cells by using SCAPS-1D. *Optoelectron. Rep.* **1**(1) (2024).

[6] Kazim S, Nazeeruddin MK, Grätzel M, Ahmad S (2014). Perovskite as light harvester: A game changer in photovoltaics. Angew. Chem. Int. Ed..

[7] Eperon GE, Stranks SD, Menelaou C, Johnston MB, Herz LM, Snaith HJ (2014). Formamidinium lead trihalide: A broadly tunable perovskite for efficient planar heterojunction solar cells. Energy Environ. Sci..

[8] Lin Q, Armin A, Nagiri RCR, Burn PL, Meredith P (2015). Electro-optics of perovskite solar cells. Nat. Photonics.

[9] Jan ST, Noman M (2023). Analyzing the effect of planar and inverted structure architecture on the properties of MAGeI3 perovskite solar cells. Energy Technol..

[10] Jan ST, Noman M (2022). Influence of absorption, energy band alignment, electric field, recombination, layer thickness, doping concentration, temperature, reflection and defect densities on MAGeI3 perovskite solar cells with Kesterite HTLs. Physica Scripta.

[11] Jan ST, Noman M (2022). Influence of layer thickness, defect density, doping concentration, interface defects, work function, working temperature and reflecting coating on lead-free perovskite solar cell. Solar Energy.

[12] Stoumpos CC, Malliakas CD, Kanatzidis MG (2013). Semiconducting tin and lead iodide perovskites with organic cations: Phase transitions, high mobilities, and near-infrared photoluminescent properties. Inorg. Chem..

[13] Wang M, Zang Z, Yang B, Hu X, Sun K, Sun L (2018). Performance improvement of perovskite solar cells through enhanced hole extraction: The role of iodide concentration gradient. Solar Energy Mater. Solar Cells.

[14] Noman M, Khan Z, Jan ST (2024). A comprehensive review on the advancements and challenges in perovskite solar cell technology. RSC Adv..

[15] van Reenen S, Kemerink M, Snaith HJ (2015). Modeling anomalous hysteresis in perovskite solar cells. J. Phys. Chem. Lett..

[16] Peng J (2017). Interface passivation using ultrathin polymer–fullerene films for high-efficiency perovskite solar cells with negligible hysteresis. Energy Environ. Sci..

[17] Polydorou E (2017). Avoiding ambient air and light induced degradation in high-efficiency polymer solar cells by the use of hydrogen-doped zinc oxide as electron extraction material. Nano Energy.

[18] McNaught AD, Wilkinson A (1997). Compendium of Chemical Terminology.

[19] Zhao P, Kim BJ, Jung HS (2018). Passivation in perovskite solar cells: A review. Mater. Today Energy.

[20] Ip AH (2015). A two-step route to planar perovskite cells exhibiting reduced hysteresis. Appl. Phys. Lett..

[21] Gil-Escrig L (2015). Efficient photovoltaic and electroluminescent perovskite devices. Chem. Commun..

[22] Wu S (2019). A chemically inert bismuth interlayer enhances long-term stability of inverted perovskite solar cells. Nat. Commun..

[23] Noel NK (2014). Enhanced photoluminescence and solar cell performance via Lewis base passivation of organic–inorganic lead halide perovskites. ACS Nano.

[24] Song D (2016). Dual function interfacial layer for highly efficient and stable lead halide perovskite solar cells. J. Mater. Chem. A.

[25] Lee JW, Seol DJ, Cho AN, Park NG (2014). High-efficiency perovskite solar cells based on the black polymorph of HC (NH2) 2PbI3. Adv. Mater..

[26] Cho KT (2017). Highly efficient perovskite solar cells with a compositionally engineered perovskite/hole transporting material interface. Energy Environ. Sci..

[27] Yoo B (2019). Improved charge separation and photovoltaic performance of BiI3 absorber layers by use of an in situ formed BiSI interlayer. ACS Appl. Energy Mater..

[28] Fu L, Nie Y, Li B, Li N, Cao B, Yin L (2019). Bismuth telluride interlayer for all-inorganic perovskite solar cells with enhanced efficiency and stability. Solar RRL.

[29] N. M. I. Center, Mineral commodity summaries 2020. In *Mineral Commodity Summaries*. National Minerals Information Center, Reston, VA, Report 2020. Accessed: 3 May. [Online]. 10.3133/mcs2020.

[30] Hu Y, Zhang S, Ruan W, Wang D, Wu Y, Xu F (2020). Interfacing pristine BiI3 onto TiO_2_ for efficient and stable planar perovskite solar cells. Appl. Surf. Sci..

[31] Fujishima A, Honda K (1972). Electrochemical photolysis of water at a semiconductor electrode. Nature.

[32] Liu D, Kelly TL (2014). Perovskite solar cells with a planar heterojunction structure prepared using room-temperature solution processing techniques. Nature Photonics.

[33] Shi D (2016). Spiro-OMeTAD single crystals: Remarkably enhanced charge-carrier transport via mesoscale ordering. Sci. Adv..

[34] Lim I (2015). Indolocarbazole based small molecules: An efficient hole transporting material for perovskite solar cells. RSC Adv..

[35] Xiong L (2018). Review on the application of SnO_2_ in perovskite solar cells. Adv. Funct. Mater..

[36] Kim G-W, Shinde DV, Park T (2015). Thickness of the hole transport layer in perovskite solar cells: Performance versus reproducibility. RSC Adv..

[37] Lei L (2018). Influence of hole transport material/metal contact interface on perovskite solar cells. Nanotechnology.

[38] Marinova N (2015). Light harvesting and charge recombination in CH_3_NH_3_PbI_3_ perovskite solar cells studied by hole transport layer thickness variation. ACS Nano.

[39] Bhattarai S, Sharma A, Muchahary D, Gogoi M, Das T (2021). Carrier transport layer free perovskite solar cell for enhancing the efficiency: A simulation study. Optik.

[40] Min H (2021). Perovskite solar cells with atomically coherent interlayers on SnO_2_ electrodes. Nature.

[41] Zhang J (2015). Bifunctional alkyl chain barriers for efficient perovskite solar cells. Chem. Commun..

[42] Dong Q (2021). Interpenetrating interfaces for efficient perovskite solar cells with high operational stability and mechanical robustness. Nat. Commun..

[43] Chen J, Seo JY, Park NG (2018). Simultaneous improvement of photovoltaic performance and stability by in situ formation of 2D perovskite at (FAPbI_3_) 0.88 (CsPbBr_3_) 0.12/CuSCN interface. Adv. Energy Mater..

[44] Noman M, Sherwani T, Jan ST, Ismail M (2023). Exploring the impact of kesterite charge transport layers on the photovoltaic properties of MAPbI 3 perovskite solar cells. Phys. Scr..

[45] Kim M (2022). Conformal quantum dot–SnO_2_ layers as electron transporters for efficient perovskite solar cells. Science.

[46] Yaowen L (2015). Multifunctional fullerene derivative for interface engineering in perovskite solar cells. J. Am. Chem. Soc..

[47] Salado M (2020). Interface engineering by thiazolium iodide passivation towards reduced thermal diffusion and performance improvement in perovskite solar cells. Adv. Funct. Mater..

[48] Li Z (2019). Spontaneous interface ion exchange: Passivating surface defects of perovskite solar cells with enhanced photovoltage. Adv. Energy Mater..

[49] Jiang Q, Zhang X, You J (2018). SnO_2_: A wonderful electron transport layer for perovskite solar cells. Small.

[50] Khadka DB, Shirai Y, Yanagida M, Miyano K (2018). Degradation of encapsulated perovskite solar cells driven by deep trap states and interfacial deterioration. J. Mater. Chem. C.

[51] Ahmad W, Noman M, TariqJan S, Khan AD (2023). Performance analysis and optimization of inverted inorganic CsGeI_3_ perovskite cells with carbon/copper charge transport materials using SCAPS-1D. R. Soc. Open Sci..

[52] Zandi S, Saxena P, Gorji NE (2020). Numerical simulation of heat distribution in RGO-contacted perovskite solar cells using COMSOL. Solar Energy.

[53] Bhattarai S, Das T (2021). Optimization of carrier transport materials for the performance enhancement of the MAGeI_3_ based perovskite solar cell. Solar Energy.

[54] Huang L (2016). Electron transport layer-free planar perovskite solar cells: Further performance enhancement perspective from device simulation. Solar Energy Mater. Solar Cells.

[55] Bhattarai S, Pandey R, Madan J, Muchahary D, Gogoi D (2022). A novel graded approach for improving the efficiency of Lead-Free perovskite solar cells. Solar Energy.

[56] Bugelman M, Nollet P, Degrave S (2000). Modeling polycrystalline semiconductors solar cells. Thin Solid Films.

[57] Afridi K, Noman M, Jan ST (2024). Evaluating the influence of novel charge transport materials on the photovoltaic properties of MASnI_3_ solar cells through SCAPS-1D modelling. R. Soc. Open Sci..

[58] Han H (2014). Defect engineering of BiI3 single crystals: Enhanced electrical and radiation performance for room temperature gamma-ray detection. J. Phys. Chem. C.

[59] Balicki M (2018). Experimental and Theoretical Optimization of BiI3 Selective-Contact Solar Cell Materials.

[60] Noman M, Shahzaib M, Jan ST, Khan Z, Ismail M, Khan AD (2024). Optimizing band gap, electron affinity, & carrier mobility for improved performance of formamidinium lead tri-iodide perovskite solar cells. Mater. Sci. Eng. B.

[61] Schulz P, Whittaker-Brooks LL, MacLeod BA, Olson DC, Loo YL, Kahn A (2015). Electronic level alignment in inverted organometal perovskite solar cells. Adv. Mater. Interfaces.

[62] Kim H, Lim K-G, Lee T-W (2016). Planar heterojunction organometal halide perovskite solar cells: Roles of interfacial layers. Energy Environ. Sci..

[63] Pandey, R. *et al.*, Halide composition engineered a non-toxic perovskite–silicon tandem solar cell with 30.7% conversion efficiency. *ACS Appl. Electron. Mater.* (2023).

[64] Karimi E, Ghorashi SMB (2017). Simulation of perovskite solar cell with P3HT hole-transporting materials. J. Nanophotonics.

[65] Bhattarai S, Pandey R, Madan J, Ahmed F, Shabnam S (2022). Performance improvement approach of all inorganic perovskite solar cell with numerical simulation. Mater. Today Commun..

[66] Ismail M, Noman M, TariqJan S, Imran M (2023). Boosting efficiency of eco-friendly perovskite solar cell through optimization of novel charge transport layers. R. Soc. Open Sci..

[67] Aneeq M, Noman M, Jan ST, Khan AD (2023). Exploring the effect of kesterites and zinc-based charge transport materials on the device performance and optoelectronic properties of FAPbI_3_ perovskite solar cells. Energy Technol..

[68] Lim KG, Ahn S, Kim H, Choi MR, Huh DH, Lee TW (2016). Self-doped conducting polymer as a hole-extraction layer in organic–inorganic hybrid perovskite solar cells. Adv. Mater. Interfaces.

[69] Bhattarai S (2023). Performance improvement of hybrid-perovskite solar cells with double active layer design using extensive simulation. Energy Fuels.

[70] Takahashi Y, Hasegawa H, Takahashi Y, Inabe T (2013). Hall mobility in tin iodide perovskite CH_3_NH_3_SnI_3_: Evidence for a doped semiconductor. J. Solid State Chem..

[71] Xiao J, Shi J, Li D, Meng Q (2015). Perovskite thin-film solar cell: Excitation in photovoltaic science. Sci. China Chem..

[72] Bhattarai S (2023). Optimized high-efficiency solar cells with dual hybrid halide perovskite absorber layers. Energy Fuels.

[73] Skoplaki E, Palyvos JA (2009). Operating temperature of photovoltaic modules: A survey of pertinent correlations. Renew. Energy.

[74] Green MA, Emery K (1993). Solar cell efficiency tables. Prog. Photovolt. Res. Appl..

[75] Grätzel M (2017). The rise of highly efficient and stable perovskite solar cells. Acc. Chem. Res..

[76] Lee JW, Kim DH, Kim HS, Seo SW, Cho SM, Park NG (2015). Formamidinium and cesium hybridization for photo-and moisture-stable perovskite solar cell. Adv. Energy Mater..

[77] Khan Z, Noman M, Tariq Jan S, DaudKhan A (2023). Systematic investigation of the impact of kesterite and zinc based charge transport layers on the device performance and optoelectronic properties of ecofriendly tin (Sn) based perovskite solar cells. Solar Energy.

[78] Park N-G, Grätzel M, Miyasaka T, Zhu K, Emery K (2016). Towards stable and commercially available perovskite solar cells. Nat. Energy.

[79] Yang Y (2016). Observation of a hot-phonon bottleneck in lead-iodide perovskites. Nat. Photonics.

[80] Noman M, Shahzaib M, Jan ST, Shah SN, Khan AD (2023). 26.48% efficient and stable FAPbI_3_ perovskite solar cells employing SrCu_2_O_2_ as hole transport layer. RSC Adv..

